# Supplementary material for: A cryo-electron microscopic approach to elucidate protein structures from human brain microsomes
Source: Life Sci Alliance. 2022 Nov 30;6(2):e202201724. doi: 10.26508/lsa.202201724 (PMC9713474; doi:10.26508/lsa.202201724)
Supplement: Supplementary file 1 [file LSA-2022-01724_TableS1.docx]

| **Table S1. Cryo-EM data collection, processing, and refinement statistics.** | | | | | |
| --- | --- | --- | --- | --- | --- |
| **Data collection** | **Peak 1 (300-500 kDa)** | | | **Peak 2 (100-300 kDa)** | |
| Magnification | 81,000 | | | 81,000 | |
| Voltage (kV) | 300 | | | 300 | |
| Electron Microscope | Krios-GIF-K3 | | | Krios-GIF-K3 | |
| Defocus (um) | -1.0 to -2.5 | | | -1.0 to -2.5 | |
| Energy filter width (eV) | 20 | | | 20 | |
| Pixel size (Å) | 1.07 (0.535) | | | 1.07 (0.535) | |
| Total dose (e^-^/Å^2^) | 35.3 | | | 36.5 | |
| Number of frames | 40 | | | 40  2,700 | |
| Number of micrographs | 2,304 | | |  |  |
| Initial particle images (no.) | 2,204,711 | | | 1,318,001 | |
| **Refinement** | DPYSL2 | GS | FT | GAPDH | ALDH1A1 |
| Number of Final Particles | 109,192 | 27,886 | 12,220 | 8,982 | 6,684 |
| Symmetry | D2 | D5 | O | D2 | D2 |
| GSFSC Resolution (Å) | 2.76 | 2.73 | 2.69 | 3.22 | 3.40 |
| **Refinement** |  |  |  |  |  |
| Initial Model (PDB code) | 5MKV | 2OJW | 2FHA | 1ZNQ | 4WJ9 |
| Model Resolution (Å) | 2.76 | 2.73 | 2.69 | 3.22 | 3.40 |
| Number of Protein Residues | 1972 | 3700 | 4128 | 1332 | 1972 |
| Number of Ligands | 0 | 10 | 24 | 0 | 0 |
| **RMSD^a^** |  |  |  |  |  |
| Bond Lengths (Å) | 0.003 | 0.002 | 0.002 | 0.003 | 0.004 |
| Bond Angles (°) | 0.562 | 0.525 | 0.413 | 0.468 | 0.71 |
| **Validation** |  |  |  |  |  |
| MolProbity Score | 1.64 | 1.06 | 1.31 | 1.54 | 2.02 |
| Clash Score | 8.07 | 2.74 | 5.73 | 7.03 | 15.10 |
| Rotamer Outliers (%) | 0.97 | 0.32 | 0.0 | 1.48 | 0.0 |
| **Ramachandran Plot (%)** |  |  |  |  |  |
| Favored (%) | 96.74 | 98.64 | 99.41 | 97.89 | 95.11 |
| Disallowed (%) | 0.00 | 0.00 | 0.00 | 0.00 | 0.00 |
| **CC Score** |  |  |  |  |  |
| Mask | 0.82 | 0.78 | 0.81 | 0.79 | 0.73 |
| Volume | 0.79 | 0.74 | 0.79 | 0.77 | 0.71 |
| Box | 0.71 | 0.64 | 0.75 | 0.67 | 0.60 |

^a^, root mean square deviation
