# Supplementary material for: A cryo-electron microscopic approach to elucidate protein structures from human brain microsomes
Source: Life Sci Alliance. 2022 Nov 30;6(2):e202201724. doi: 10.26508/lsa.202201724 (PMC9713474; doi:10.26508/lsa.202201724)
Supplement: Supplementary file 2 [file LSA-2022-01724_TableS2.docx]

**Table S2A. Brain Tissue Protein (300-500 kDa)**

| **Hit** | **Score** | **Coverage** | **Mass (Avg, Da)** | **Peptides Identified** | **Spectral Matches** | **Description** |
| --- | --- | --- | --- | --- | --- | --- |
| **1** | **1634** | **66%** | **74072.948** | **38** | **187** | **Human Dihydropyrimidinase related protein 2** |
| 2 | 1564 | 70% | 62710.74 | 34 | 184 | Human Dihydropyrimidinase related protein 3 |
| 3 | 1482 | 70% | 62124.81 | 46 | 124 | Human Pyruvate kinase PKLR |
| 4 | 1455 | 62% | 85058.5 | 54 | 104 | Human Heat shock protein HSP 90-alpha |
| 5 | 1408 | 78% | 53558.277 | 43 | 120 | Human Pyruvate kinase PKM |
| 6 | 1311 | 46% | 193381.948 | 71 | 136 | Human Clathrin heavy chain |
| 7 | 1047 | 58% | 49936.915 | 40 | 67 | Human Glial fibrillary acidic protein |
| 8 | 1032 | 62% | 71125.788 | 38 | 63 | Human Heat Shock Cognate 71 kDa |
| …. |  |  |  |  |  |  |
| **16** | **695** | **42%** | **42691.759** | **23** | **50** | **Human Glutamine Synthetase** |
| …. |  |  |  |  |  |  |
| 36 | 478 | 54% | 61991.45 | 22 | 33 | Human Dihydropyrimidinase related protein 5 |
| …. |  |  |  |  |  |  |
| **61** | **331** | **55%** | **21383.36** | **10** | **40** | **Human Ferritin Heavy Chain** |

**Table S2B. Brain Tissue Protein (100-300 kDa)**

| **Hit** | **Score** | **Coverage** | **Mass (Avg, Da)** | **Peptides Identified** | **Spectral Matches** | **Description** |
| --- | --- | --- | --- | --- | --- | --- |
| 1 | 1930 | 73% | 71317.247 | 48 | 181 | Human Serum Albumin |
| 2 | 1482 | 84% | 47481.45 | 33 | 180 | Human Alpha-enolase |
| 3 | 1351 | 85% | 47581.13 | 31 | 174 | Human Gamma-enolase |
| 4 | 1237 | 95% | 39876.16 | 31 | 106 | Human Fructose-bisphosphate aldolase A |
| 5 | 1188 | 81% | 39854.95 | 31 | 88 | Human Fructose-bisphosphate aldolase C |
| **6** | **1173** | **77%** | **36201.46** | **25** | **164** | **Human Glyceraldehyde-3-phosphate dehydrogenase** |
| 7 | 971 | 61% | 68518.98 | 30 | 66 | Human Transketolase |
| 8 | 950 | 59% | 58470.24 | 29 | 76 | Human Pyruvate Kinase |
| …. |  |  |  |  |  |  |
| **12** | **779** | **59%** | **56858.82** | **28** | **38** | **Human Aldehyde Dehydrogenase A1** |
